# Baseline-free structured light 3D imaging using a metasurface double-helix dot projector

**DOI:** 10.1515/nanoph-2024-0668

**Published:** 2025-02-07

**Authors:** Zicheng Shen, Yibo Ni, Yuanmu Yang

**Affiliations:** State Key Laboratory of Precision Measurement Technology and Instruments, Department of Precision Instrument, 12442 Tsinghua University, Beijing 100084, China

**Keywords:** structured light 3D imaging, baseline-free, metasurface double-helix dot projector

## Abstract

Structured light is a widely used 3D imaging method with a drawback that it typically requires a long baseline length between the laser projector and the camera sensor, which hinders its utilization in space-constrained scenarios. On the other hand, the application of passive 3D imaging methods, such as depth from depth-dependent point spread functions (PSFs), is impeded by the challenge in measuring textureless scenes. Here, we combine the advantages of both structured light and depth-dependent PSFs and propose a baseline-free structured light 3D imaging system. A metasurface is designed to project a structured dot array and encode depth information in the double-helix pattern of each dot simultaneously. Combined with a straightforward and fast algorithm, we demonstrate accurate 3D point cloud acquisition for various real-world scenarios including multiple cardboard boxes and a living human face. Such a technique may find application in a broad range of areas including consumer electronics and precision metrology.

## 1 Introduction

Three-dimensional (3D) imaging technology, due to its pivotal role in enabling machines and artificial intelligence to perceive and interact with the world, has drawn enormous interest in recent years [1], [2], [3], [4], [5]. Structured light, as one of the most commonly adopted 3D imaging technologies [6], can allow highly reliable acquisition of 3D point clouds and has found extensive applications in emerging fields such as consumer electronics and robotics. Structured light technology relies on the triangulation principle to measure depth, which consequently necessitates a baseline length between the laser projector and the camera sensor and oftentimes requires a complicated image-matching algorithm [7], [8]. The baseline length between the projector and receiver leads to bulky hardware. For instance, smartphones equipped with structured light-based 3D imaging modules typically have multiple black openings or a long black stripe on their screens. Moreover, an extremely long baseline length is required for high-accuracy 3D imaging at long distances. The matching algorithm, typically including calibration, image correlation, cost aggregation, and depth calculation, imposes a high demand on computational resources [9], [10].

Depth-from-defocus (DfD) is another widely studied 3D imaging method, since it is not limited by the principle of triangulation and can obtain depth information from axial image blur using only a single camera [11], [12], [13], [14], [15]. More sophisticated depth-dependent point-spread functions (PSFs) are also proposed to further improve the depth accuracy of DfD, such as double-helix PSF, which features two foci rotating around a central point with the rotation angle depending on the axial depth of the object point [16], [17], [18], [19], [20], [21], [22]. Nevertheless, since its depth calculation relies on the texture of the target object, DfD often fails in measuring textureless scenes. In addition, DfD typically requires the use of relatively complex image feature extraction and matching algorithms, resulting in even higher computational costs. A 3D imaging system combining the high reliability of conventional structured light techniques and the compactness of the DfD method is highly desired.

Optical metasurfaces [23], [24], [25], [26], [27], [28], [29], [30], [31], [32], [33], [34], due to their versatility in tailoring the light field at subwavelength scale, have been widely adopt to remarkably enhance the performance and compactness of 3D imaging systems [19], [35], [36], [37], [38], [39], [40], [41], [42], [43]. Notably, Li et al. recently proposed a structured light-based 3D imaging method without triangulation based on 3D holograms using metasurface [44]. By eliminating the need for baseline length, the volume of structured light systems can be reduced. Nonetheless, since the light intensity distribution of 3D holograms is only considered at a few discrete distances, clear and continuous correspondence between distance and hologram distribution is absent. In addition, the image correlation algorithm is still rather complicated. It remains a major challenge to build a compact structured light-based 3D imaging system that allows high-accuracy depth sensing for continuous depth values using a fast algorithm.

Here, we propose and experimentally demonstrate accurate 3D point cloud generation for various complex scenes using a compact baseline-free structured light system equipped with a metasurface double-helix dot projector. Leveraging the versatility of metasurface to manipulate the light field at subwavelength precision, we design and optimize a metasurface projector that projects a structured dot array and encodes depth information in the double-helix pattern of each dot simultaneously. The rotation angle of the double-helix pattern of each dot has a continuous monotonic one-to-one correspondence relationship with depth. A beam splitter is employed to fold the projection and receiving light path to enable a single-opening system configuration. Combined with a straightforward and fast algorithm to calculate depth from the rotation angle of the dot patterns, we demonstrate accurate 3D point cloud acquisition for scenes including multiple boxes and a living human face, towards applications including robotic operation and 3D face authentication.

## 2 Results and discussions

### 2.1 Framework of the baseline-free structured light 3D imaging system

**Figure 1:**
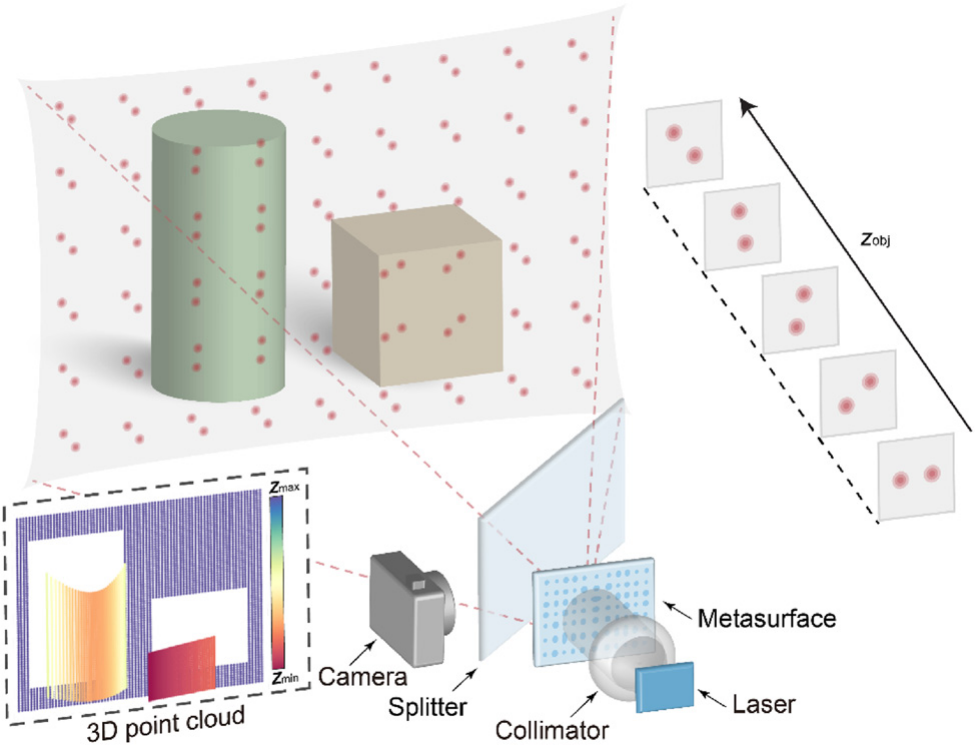
Schematic of baseline-free structured light 3D imaging system using a metasurface double-helix dot projector. The light source of the projector is a collimated laser, after being modulated by the metasurface, a dot array with depth-related double-helix patterns is projected. The rotation angle of each projected double-helix pattern can be mapped to the depth value *z*_obj_, thus eliminating the baseline length requirement in a traditional structured light system. The receiver is a conventional camera that captures the structured light pattern. We use a beam splitter to fold the projection and receiving light path and align the center of the camera imaging plane with the center of the projector. Therefore, the projector and receiver share a single opening. From a single image captured by the camera, one can reconstruct an accurate 3D point cloud of the scene.

The framework of the baseline-free structured light 3D imaging system is schematically illustrated in Figure 1. At the projection end, with a collimated 635-nm laser as the light source, a metasurface is designed and optimized to project a 64 × 64 dot array with a 60° diagonal field-of-view (FOV), while encoding depth information in the double-helix pattern of each dot. The receiving end consists of a camera with a sufficient FOV and high resolution. The optical axes of the dot projector and the camera are perpendicular to each other, with a beam splitter adopted to fold the projection and receiving light paths and align the center of the camera imaging plane with that of the projector. Consequently, the projector and receiver share a single opening. From a single image captured by the camera, based on the one-to-one correspondence between the rotation angle of the double-helix pattern and the object depth, an accurate 3D point cloud of the scene can be generated.

### 2.2 Metasurface design and fabrication

To realize a dot projector with a depth-dependent double-helix pattern, we first need to determine the transmission phase distribution of the metasurface. The phase to generate a rotating double-helix pattern is initialized by arranging generalized Fresnel zones carrying spiral phase profiles with gradually increasing topological quantum numbers towards the outer rings of the zone plate [45], [46]. The phase term $\psi \left(u,\varphi_{u}\right)$ is given by,

(1) $$\begin{array}{ll} \psi \left(u,\varphi_{u}\right) & =\left\{\left[\left(l-1\right)\times 2+1\right]\left.\varphi_{u}\right|{\left(\frac{l-1}{L}\right)}^{\varepsilon }\right.\leq u\leq {\left(\frac{l}{L}\right)}^{\varepsilon },\\ & \;\left.l=1,\ldots ,L\right\}, \end{array}$$

where *u* is the normalized radial coordinate and *φ_u_* is the azimuth angle in the aperture plane. $\left[L,\varepsilon \right]$ are adjustable design parameters**.** Here we choose $\left[L,\varepsilon \right]=\left[12,0.8\right]$ as our initial phase design to generate double-helix patterns with suitable distance between the two main lobes. Compared with the Gauss–Laguerre mode-based approach widely used in the design of double-helix PSFs [19], [36], [47], the adopted generalized Fresnel zone approach can generate a more compact rotating pattern with the shape of the pattern kept almost invariant over an extended depth of field [45].

**Figure 2:**
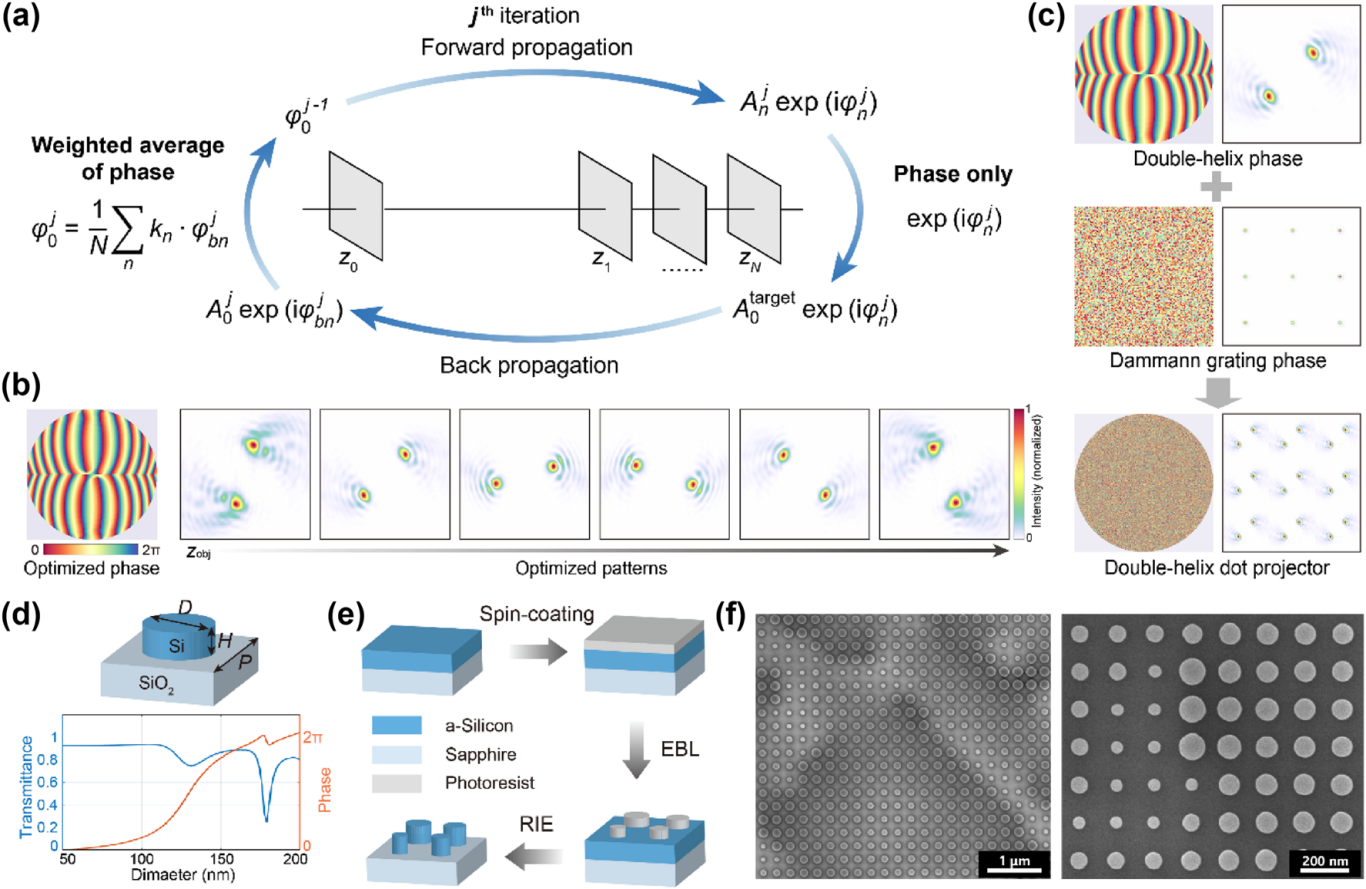
Metasurface design and fabrication. (a) Optimization process of the metasurface phase profile. The forward propagation and inverse propagation are calculated using the angular spectrum method. *z*_0_ is the location of the metasurface plane, and *z*_1_ ∼ *z*_*N*_ are different propagation distances. $\varphi_{0}^{j}$ is the double-helix pattern encoding phase after *j*th iteration. $A_{n}^{j}\exp\left(i\varphi_{n}^{j}\right)$ is the complex amplitude of the light field propagated to distance *z*_*n*_. $A_{0}^{\text{targrt}}$ is the target amplitude distribution of the double-helix pattern. $A_{0}^{j}\exp\left(i\varphi_{bn}^{j}\right)$ is the complex amplitude of the light field back propagated to the metasurface plane from distance *z*_*n*_. *k*_*n*_ is the weight of the weighted average of phase. (b) Optimized pattern encoding phase profile and numerically calculated pattern as a function of the projected depth. (c) Phase design of the metasurface. The phase profile of the metasurface is the linear superposition of the Dammann grating phase which forms a dot array and the double-helix phase which tailors the pattern of each dot. (d) The unit-cell of the metasurface is composed of silicon nanopillars with circular in-plane cross-sections on a sapphire substrate, with height *H* = 300 nm, period *U* = 250 nm, the diameter *D* is swept between 50 and 200 nm to achieve a full 2π phase modulation and a high transmittance. (e) Fabrication process of the metasurface. (f) Scanning electron microscopy images of the fabricated metasurface sample.

Subsequently, an iterative Fourier transform algorithm is employed to maximize the energy in the main lobe of the double-helix pattern within the 180° rotation range, as shown in Figure 2a. The iterative optimization process improves the peak intensity of the main lobe of the double-helix pattern by 31 % in average. The optimized phase to generate double-helix patterns and the corresponding patterns at different projection distances are shown in Figure 2b. To avoid ambiguities, the double-helix patterns beyond the 180° rotation range are designed to spread significantly, and can be easily distinguished from those within the 180° rotation range.

Meanwhile, a Dammann grating phase that projects a highly uniform 64 × 64 normal structured light dot array over the 60° diagonal FOV is designed using the Gerchberg–Saxton algorithm [48]. Through the superposition of the double-helix pattern encoding phase profile and the Dammann grating phase profile, we obtain the target phase profile of the metasurface that projects a double-helix dot array, as shown in Figure 2c.

The unit cell of the metasurface is composed of silicon nano-cylinder (top panel of Figure 2d), with a height *H* = 300 nm, and a period *U* = 250 nm. The diameter *D* of the nano-cylinder is swept between 50 and 200 nm to achieve a full 2π phase modulation and a transmittance above 78 % (bottom panel of Figure 2d). As shown in Figure 2e, the fabrication of the metasurface starts with spin-coating of photoresist on a silicon-on-sapphire substrate, where the thickness of the monocrystalline silicon film is 300 nm. Electron beam lithography (EBL) is employed to write the metasurface pattern onto the photoresist. Subsequently, the pattern is transferred to the silicon layer via reactive ion etching (RIE). Finally, the photoresist layer is removed. The scanning electron microscopy images of the fabricated metasurface are shown in Figure 2f.

### 2.3 Experimental system setup

**Figure 3:**
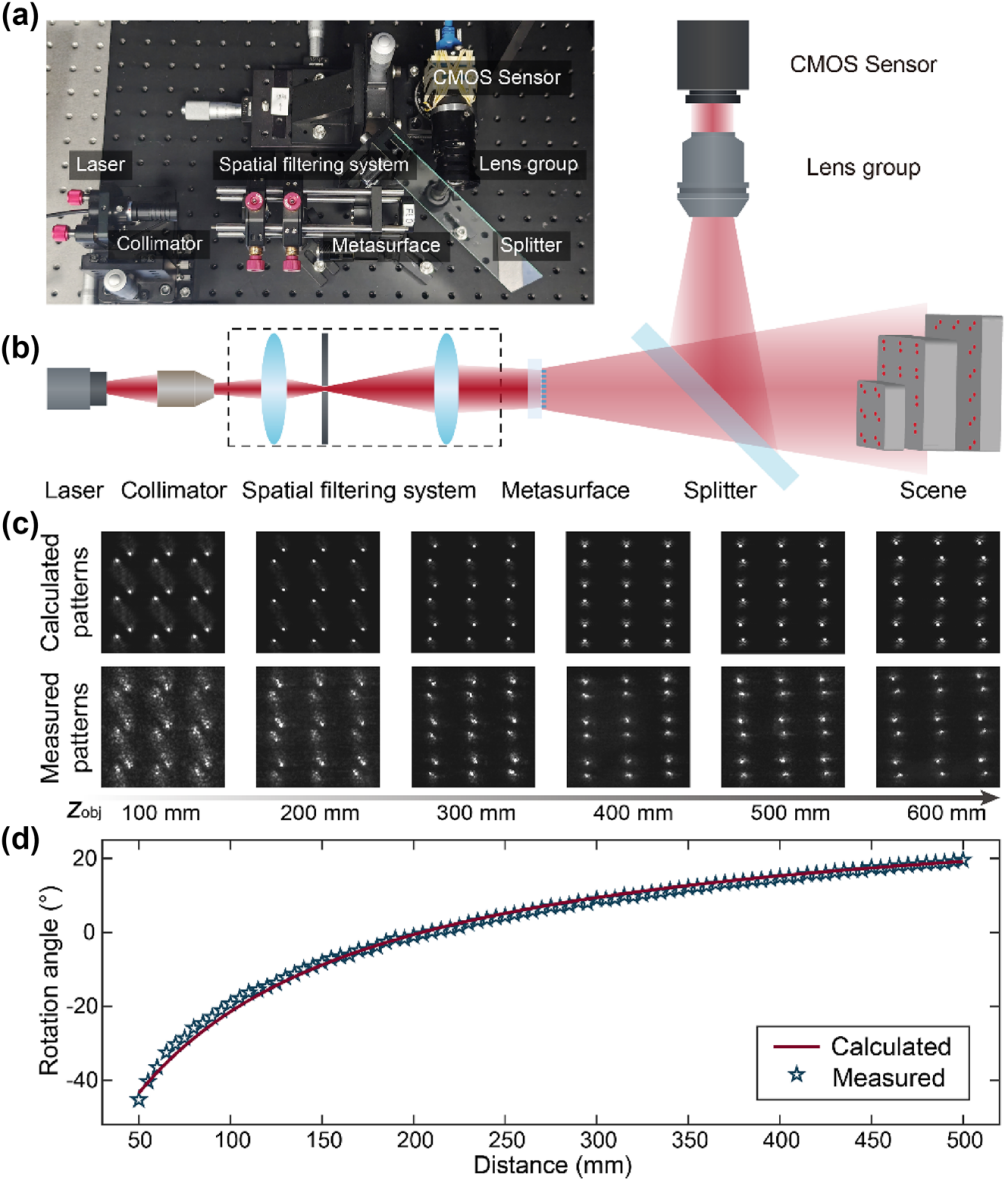
Experimental system and calibration. (a) Photograph of the experimental set-up. (b) Schematic of the experimental set-up. (c) Calculated (top panel) and experimentally measured (bottom panel) patterns of the central area of the dot array. (d) Calculated (red line) and experimentally measured (blue star) rotation angles of the projected pattern as a function of the distance.

The photograph of the experimental setup is shown in Figure 3a. As schematically illustrated in Figure 3b, the light source of the experimental system is a 635-nm diode laser (SZ Laser ZLMAD635-16GD). After being collimated, the laser passes through a spatial filtering system consisting of two lenses and a pinhole, which is used to improve the laser beam quality, and is incident on the metasurface. Although the relative positions of the projector and the camera in the structured light system can be arbitrarily arranged, we use a beam splitter to fold the projection and receiving light paths and align the center of the camera’s imaging plane and the center of the metasurface at the projection end so that they completely overlap in their corresponding mirror space. Thereby, we can realize a single-opening system configuration. Moreover, since the central position of each dot is fixed in the captured image, the depth reconstruction process can be drastically simplified and accelerated. The camera is equipped with a CMOS image sensor (Daheng Imaging ME2P – 2622 – 15U3M) with an active area of 12.8 × 12.8 mm^2^ and a 12-mm focal length lens group with a low image distortion.

The correspondence between distances and rotation angles of the double helix pattern is calibrated before 3D imaging of actual scenes. We place a flat plate at different distances to measure the distributions of the projected double helix dot array. The comparison between central areas of the measured and calculated double helix dot arrays is shown in Figure 3c. Figure 3d shows that the calculated and experimental measured relationships between distances and the rotation angles of the double helix pattern are in close agreement.

### 2.4 Experimental demonstration of 3D imaging

**Figure 4:**
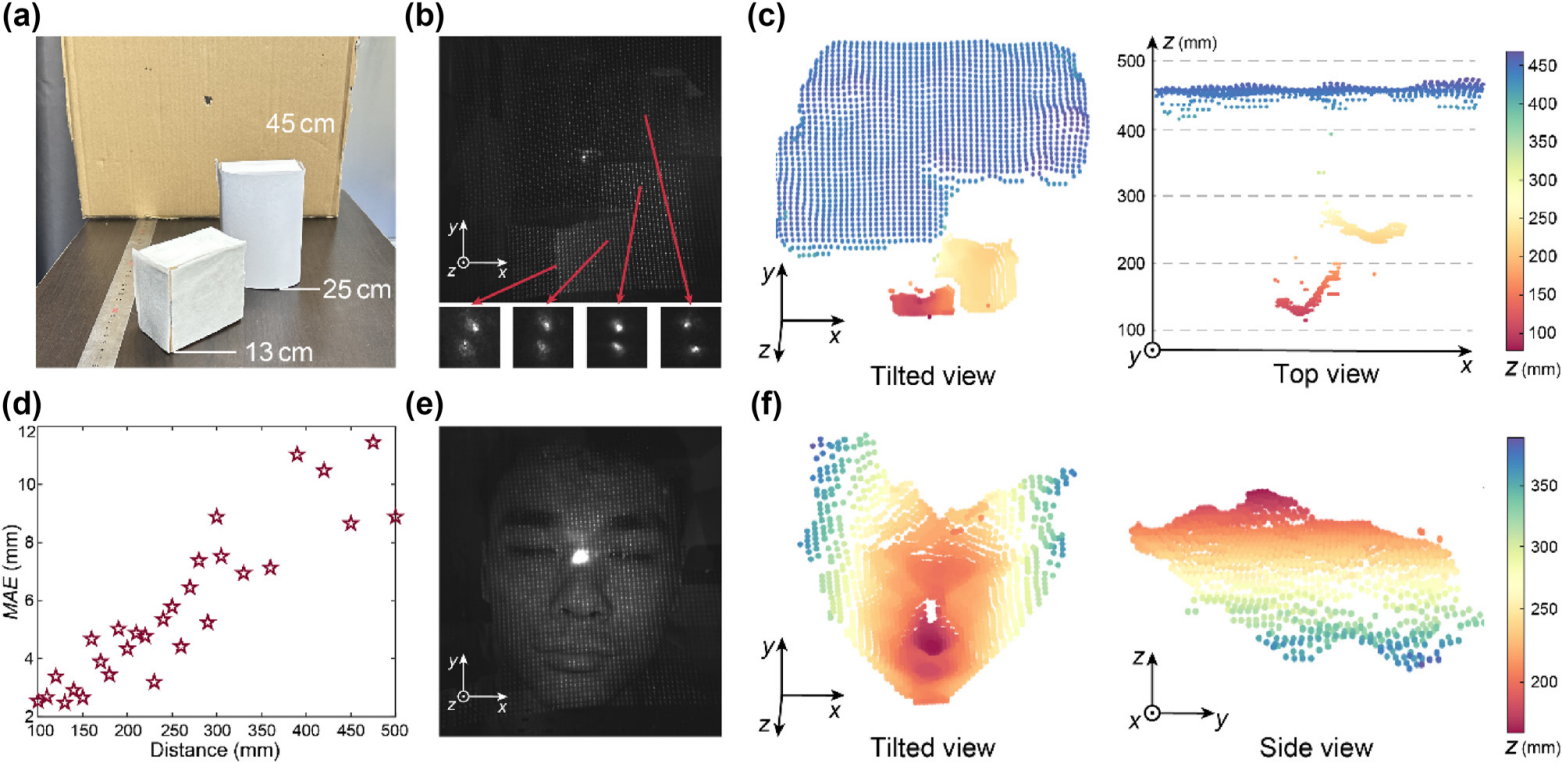
Experimental demonstration of 3D imaging. (a) Photograph of a target scene consisted of three cardboard boxes. (b) The raw image captured by the proposed structured light system for the scene shown in panel (a). The red arrows point to the regions of the magnified dot patterns. (c) Tilted view (left panel) and top view (right panel) of the 3D point cloud of the scene shown in panel (a) generated by the baseline-free structured light 3D imaging system. (d) Quantitative test result of depth accuracy. The red stars are the measured mean absolute error (*MAE*) at each distance. (e) Raw image captured by our structured light system for a living human face (the face of the 1st-author). (f) Tilted view (left panel) and side view (right panel) of the 3D point cloud of the scene shown in panel (e) generated by the baseline-free structured light 3D imaging system.

To demonstrate 3D imaging of real-world scenarios, we first set up a scene consisting of three cardboard boxes located at different distances, a scenario that may occur in industrial robotic operations, as shown in Figure 4a. Figure 4b shows the raw image captured by the proposed system. In the inset of Figure 4b, four double helix patterns at different distances are magnified, showcasing differences in their rotation angles. Based on the experimentally calibrated relationship between the rotation angles of the double helix patterns and distances and a straightforward algorithm that calculates the rotation angle of the double helix pattern of each dot, a 3D point cloud can be generated within 0.4 s from the raw measurement, on a laptop computer with Intel i7-10875H CPU and 16 GB RAM. As shown in Figure 4c, the calculated 3D point cloud accurately depicts the 3D distribution of the scene, including the individual shapes and precise absolute distances of each box. There are some less accurate stray points in the 3D point cloud, which could potentially be minimized by enhancing the signal-to-noise ratio of the imaging system and by optimizing the 3D reconstruction algorithm. The reconstruction can also be further accelerated when deploying to edge computation platforms, through operator optimization or hardware acceleration [49].

To quantitatively assess the depth measurement accuracy of the baseline-free structured light 3D imaging system, we deploy a flat cardboard plate at different distances and calculate the mean absolute error (MAE) between the measured depth value of each dot and the true depth value to characterize the distance measurement error. As shown in Figure 4d, the depth measurement errors are predominantly below 1 cm for all distances within the depth measuring range of 500 mm. We further calculate the relative depth measurement error, defined as distance measurement error Δ*z*_obj_ divided by true distance *z*_obj_, of our system. The calculated relative depth measurement error of our system is about 2.4 %, which is close to the relative depth measurement error of within 2 % of typical commercial structured light products, such as Intel^®^ RealSense™ Depth Camera D435.

A single-opening structured light system is ideally suited for space-constrained platforms. Here, 3D reconstruction of a living human face, which is a widely used function on smartphones, is demonstrated using the proposed system. We capture an image of a living human face located at distances between 20 and 40 cm, as shown in Figure 4e. The resulting 3D point cloud exhibits fine features of the human face, as shown in Figure 4f. Note that the zero-order light spot in Figure 4e has a measured power of less than 0.01 mW, which complies with the laser safety standard for human eyes [50], and could be substantially diminished by optimizing the fabrication process of the metasurface.

## 3 Conclusions

In summary, we have demonstrated a baseline-free structured light system for accurate and rapid 3D imaging of different real-world scenarios. By exploiting the subwavelength precision light field manipulation ability of metasurfaces for the projection of double-helix dot array, high-accuracy 3D imaging for complex scenes using a baseline-free structured light system is achieved.

The single-opening configuration enabled by the folded light path, which can be substantially shrunk in volume via the standard lens module assembly process [51], holds potential in various application scenarios that require miniaturization of the 3D imaging system, such as 3D face authentication of smartphones, robotic operations, and endoscopes. For application scenarios that are highly sensitive to the thickness of the 3D imaging system, such as smartphones, we envision that by adopting a 45° prism mirror that has been widely applied in periscope telephoto modules on smartphones [52] to rotate the optics by 90° in the housing, the thickness of the baseline-free single-opening structured light system may be further reduced.

The straightforward depth calculation algorithm, simpler than those of existing structured light techniques, may empower high frame-rate 3D imaging on various mobile platforms that can only carry limited computing resources. For applications requiring larger FOV, by using near-to-far field transformation methods that do not rely on the paraxial approximation in the Dammann grating phase design process, the FOV of our system may be expanded up to 180° [41], [53].

Similar to the effect of increasing baseline length *L* in triangulation-based 3D structured light systems, the increase of the diameter *D* of the metasurface projector in our baseline-free system can be used to increase the depth measurement accuracy and range. For instance, to maintain the same relative depth measurement error Δ*z*_obj_/*z*_obj_ for 100 times further distance, triangulation-based 3D structured light systems need 100 times larger baseline length *L*, while our baseline-free system needs 10 times larger diameter *D* of the metasurface projector [21]. Apart from diameter *D* of the metasurface projector, the relatively low signal-to-noise ratio (SNR) of the captured image and the fabrication error of the metasurface are the other two factors that restrict the current accuracy of our system and can be further improved in the future engineering implementation process. We anticipate that by applying a high-quality laser source with higher power to improve the SNR of our captured image, refining our metasurface fabrication process, as well as optimizing the metasurface design for better fabrication error tolerance, further improvement of our depth measurement accuracy can be realized. Such improvements in the 3D imaging range and accuracy of our system may help it to be adapted to an even broader range of application scenarios. Such a compact, accurate, and reliable 3D imaging solution may be useful for numerous application domains, including but not limited to consumer electronics, robot vision, autonomous driving, and biomedical imaging.
